# Atypical parkinsonian syndromes in a North African tertiary referral center

**DOI:** 10.1002/brb3.1924

**Published:** 2020-11-11

**Authors:** Amina Nasri, Mouna Ben Djebara, Ikram Sghaier, Saloua Mrabet, Sabrina Zidi, Amina Gargouri, Imen Kacem, Riadh Gouider

**Affiliations:** ^1^ Neurology Department, LR18SP03, Clinical Investigation Center (CIC) "Neurosciences and Mental Health" Razi University Hospital Tunis Tunisia; ^2^ Faculty of Medicine of Tunis University of Tunis El Manar Tunis Tunisia

**Keywords:** atypical parkinsonism, epidemiology, Parkinson's disease

## Abstract

**Introduction:**

Data on epidemiology of atypical parkinsonian syndromes (APS) in North African countries are limited. Our objective was to study the epidemiological features of APS in a Tunisian population.

**Methods:**

We conducted a 17‐year retrospective cross‐sectional descriptive study in the Department of Neurology at Razi University Hospital. We included all patients responding to consensus diagnosis criteria of APS. We recorded demographic and clinical data. Group differences were assessed with a post hoc ANOVA with a Bonferroni error correction.

**Results:**

We included 464 APS patients. Hospital prevalence of APS among all parkinsonism cases was 20.6%. Mean annual increase of incidence defined as newly diagnosed APS cases per year reached 38.8%/year. APS were divided into 4 etiological subgroups: dementia with Lewy bodies (DLB; 56.7%); progressive supranuclear palsy(PSP; 16.2%); multiple system atrophy (MSA; 14.6%); and finally corticobasal syndrome (CBS; 12.5%). Sex‐ratio was 1.2. This male predominance was found in all subgroups except MSA (*p* = .013). Mean age at onset was 68.5 years, most belated in DLB (69.7 years; *p* < .001). Young‐onset parkinsonism (<40 years) was found only in MSA subgroup (*p* = .031). Parkinsonism was of late onset (>70 years) in 50.7% of patients and was significantly associated with DLB subgroup (*p* = .013). Inaugural parkinsonism was associated with CBS and MSA (*p* = .0497), and gait disorders at disease onset were associated with PSP and MSA (*p* = .0062). Cognitive and mood disorders were more marked in DLB and most preserved in MSA. Consanguinity was more marked in CBS (*p* = .037), and family history of dementia and psychiatric diseases was more common in DLB. Thirty‐seven families with similar cases of APS were identified.

**Conclusions:**

This is the largest African epidemiological study on APS. In our population, APS were frequent and dominated by DLB. The age of onset of parkinsonism was the most decisive feature for differential diagnosis.

## Graphical abstract

In this paper, we described the largest cohort of atypical parkinsonian syndromes (APS) ever reported in both sub‐Saharan and North African countries comprising 464 Tunisian patients with APS. In our population, APS were frequent and dominated by DLB. The age of onset of parkinsonian syndrome was the most decisive feature for differential diagnosis.

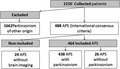

## 1 INTRODUCTION

Atypical Parkinsonian syndromes (APS) or “Parkinson‐plus” syndromes describe a spectrum of heterogeneous neurodegenerative disorders. Although the term “atypical” supposes the existence of additional atypical signs and symptoms for Parkinson's Disease (PD), only multiple system atrophy (MSA), progressive supranuclear palsy (PSP), and corticobasal degeneration (CBD) are commonly included under this umbrella term (Mulroy et al., 2019; Stamelou & Bhatia, 2016). Nevertheless, the nosology range of these disorders is relentlessly expanding. In addition to the cited pathologies, APS could include dementia with Lewy bodies (DLB) (Deutschländer et al., 2018) DLB and MSA belong to the subgroup of synucleinopathies, whereas CBD and PSP are part of the subgroup of tauopathies (Foguem & Kamsu‐Foguem, 2016; Stamelou & Bhatia, 2016). In this context of diagnosis and nosology difficulties, the study of the epidemiology of APS can be challenging. Given the rapid growth of the elderly population in developing countries, like in Tunisia (Hussein & Ismail, 2017), and its consequences for national healthcare systems, epidemiology of APS in these regions should be investigated in greater depth. To determine the epidemiologic features of APS in a Tunisian population, we conducted a retrospective cross‐sectional descriptive study and analyzed their hospital prevalence and incidence and the demographic characteristics of the different subgroups of APS.

## 2 MATERIALS AND METHODS

### 2.1 Investigation protocol

A retrospective cross‐sectional descriptive study was carried in the Department of Neurology at Razi Hospital, a tertiary care hospital in Tunis in North Tunisia, including all patients responding to the respective international consensus diagnosis criteria one of the following APS, that is, DLB (McKeith et al., 2017), MSA (Gilman et al., 2008), PSP (Höglinger et al., 2017) and corticobasal syndrome (CBS) (Armstrong et al., 2013), over a period of 17 years (from 2003 to 2019). Considering the initial hospital clinic diagnosis, we retrospectively applied the diagnosis criteria of APS to collected cases, comprising patients either initially classified as having parkinsonism or those initially classified as having one of the APS without parkinsonism. Patients were than reclassified accordingly. All patients had a neurological examination performed by a movement disorder specialist. Data were collected from the primary medical records and/or during patient's visit. We retrospectively reviewed the charts of all included patients with a reconstitution of their demographic data, family, personal and clinical history, complete physical, and neurological examination. The type and age of occurrence of initial motor (parkinsonism (tremor or akinesia), gait disorders, falls, movement disorders) and nonmotor (cognitive, behavioral, autonomic, and sleep disorders) symptoms were specified. The age of onset of the disease was defined as the age of occurrence of initial motor and/or nonmotor symptoms. The age of onset of parkinsonism was specified whether occurring at disease onset or later. The diagnostic delay was defined as the duration between the age at disease onset and the age at diagnosis. Neuropsychological assessment, performed at first consultation, comprised the Mini Mental State Examination (MMSE), the Frontal Assessment Battery (FAB), the Geriatric Depression Scale (GDS) (in patients of >65 years), or the Beck Depression Inventory (BDI) (in patients of <65 years) and the Neuropsychiatric Inventory (NPI).

Systematic morphological brain imaging (CT scan and/or MRI) was used on an exclusionary basis to rule out other CNS diseases, excluding space‐occupying lesions and to assess patterns of atrophy that aids diagnoses made by neurological assessment. This avoids the inclusion of other possible etiologies mimicking APS presentations such as brain tumors (Morelli et al., 2014), vascular lesions (Koga et al., 2019), or Creutzfeldt Jakob disease (Arnao et al., 2015). All patients with no brain imaging were not included. This group of patients was classified as APS of non established diagnosis.

We excluded all patients with Parkinson's disease (PD) according to the UK Parkinson's Disease Society Brain Bank (UKPDSBB) criteria or with parkinsonian syndrome of secondary origin (drug‐induced, vascular, infectious, metabolic, tumor, post‐traumatic, normal pressure hydrocephalus). Included patients were classified in the following subgroups: DLB, MSA, PSP, and CBS. For PSP patients, we also specified the phenotype according to the 2017 MDS criteria (Höglinger et al., 2017). For MSA, based on initial presentation, we divided the patients into three subgroups: the parkinsonian subtype (MSA‐P), the cerebellar subtype (MSA‐C), and finally a group of MSA patients with mixed phenotype.

### 2.2 Data base and statistical analysis

Continuous variables were expressed as mean ± standard deviation. Fisher's exact probability test for frequency tables was used for statistical analysis. Chi‐square test was used for categorical variables and *t* test to compare continuous variables (means) between subgroups. A post hoc ANOVA (corrected for multiple comparisons with a Bonferroni error correction) was performed to analyze significant differences between the groups. A value of *p* < .05 was considered statistically significant. Statistical analyses were performed using SPSS software.

### 2.3 Ethics

The patients gave their written informed consent, and the study was approved by the local ethics committee in Razi Hospital. All data were anonymized to protect privacy and confidentiality. The study was conducted according to guidelines of declaration of Helsinki.

## 3 RESULTS

**FIGURE 1:**
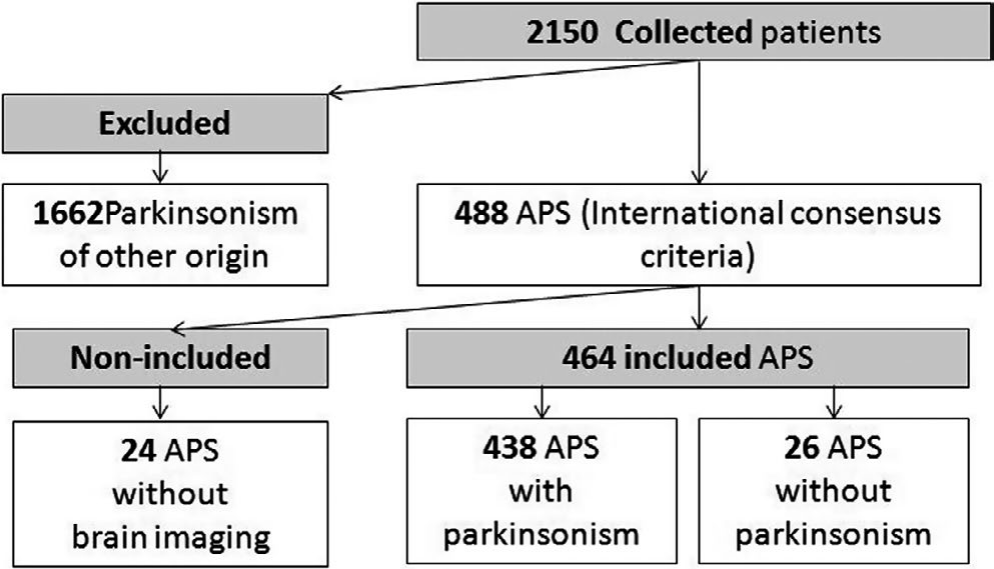
Flowchart of the study. APS, Atypical parkinsonian syndrome

Among the 2,150 collected patients during the 17‐year period of the study, diagnosed either with parkinsonian syndrome or APS without parkinsonism (for instance, patients with cerebellar form of MSA or patients with DLB who have not yet developed parkinsonism until the date of inclusion), 488 responded to the international consensus criteria of at least one of the APS. Among them, only 464 patients had brain imaging and were included for this study. Twenty‐six patients did not yet develop parkinsonism during the study period. Hence, the hospital prevalence of APS among all parkinsonism cases was 20.6%. A flow diagram of the study is depicted in Figure 1.

The annual incidence in our department increased from 5 patients in 2003 to 43 new cases in 2019, with a peak of incidence of 54 new cases per year, corresponding to a mean annual increment of 38.8% per year. In the same period, the mean annual increment of the number of all new consulting outpatients in our department was 7.8%.

**FIGURE 2:**
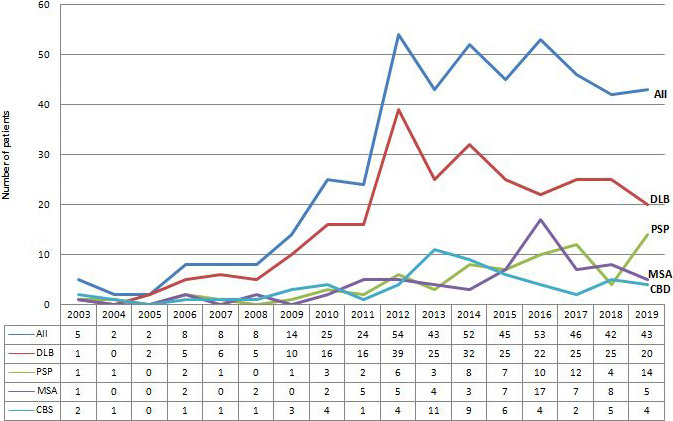
Annual number of patients with subgroups of atypical parkinsonian syndromes according to the year of first consultation. CBS, Corticobasal syndrome; DLB, Dementia with Lewy bodies; MSA, Multiple system atrophy; PSP, Progressive supranuclear palsy

The most frequent subtype of APS in our cohort was DLB (56.7%; *n* = 263), followed by PSP (16.2%; *n* = 75), MSA (14.6%; *n* = 68), and CBS (12.5%; *n* = 58). This distribution remained broadly steady during the period of the study (Figure 2).

For the PSP subgroup, the main phenotype was the typical Richardson's syndrome (PSP‐RS) (34.7%; *n* = 26), followed by PSP‐CBS (17.3%; *n* = 13) than PSP with predominant parkinsonism (PSP‐P) (12%; *n* = 9), speech/language disorder (PSP‐SL) (9.3%; *n* = 7), postural instability (PSP‐PI) (9.3%; *n* = 7), frontal presentation (PSP‐F) (8%; *n* = 6), ocular motor dysfunction (PSP‐OM) (4%; *n* = 3), with progressive gait freezing (PSP‐PGF) (2.7%; *n* = 2), primary lateral sclerosis (PSP‐PLS) (1.3%; *n* = 1), and cerebellar ataxia (PSP‐C) (1.3%; *n* = 1). For MSA patients, the MSA‐P subtype was the most common reaching 73.5% (*n* = 50) and the MSA‐C subtype the least frequent one with 10.3% (*n* = 7). A mixed phenotype was found in 16.2% (*n* = 11).

In our cohort, significant differences in sex‐ratio (SR) were observed (*p* = .013). The SR overall was 1.17 with male predominance among DLB, PSP, and CBS subgroups (1.22, 1.64, and 1.23, respectively). Interestingly, female predominance (SR = 0.61) was found among MSA patients. The overall mean age at onset was 68.5 ± 10.8 years, while the age of diagnosis was 71.76 ± 11.2 with average diagnostic delay of 3.4 ± 3.8 years. Significant differences in mean age of onset were noticed across APS subgroups *p* < .001. In fact, the most belated was found in DLB patients (73.79 ± 8.72 years) and earlier in MSA (58.85 ± 9.7 years). Among all APS, only in DLB onset occurred beyond the age of 85 years, and there was a significant association in DLB subgroup with an onset beyond the age of 65 years (85.17% of DLB patients, *p* < .001). In contrast, early onset before the age of 65 years was more commonly noted in MSA patients (70.41%) (Table 1).

**TABLE 1:** Patient's characteristics

Explanatory variables	APS *N* = 464	DLB *N* = 263	PSP *N* = 75	MSA *N* = 68	CBS *N* = 58	*p* value*
Mean annual increment of incidence (per year)	38.8%	31.4%	47.9%	39.9%	36%	—
Sex‐ratio (Male/Female)	1.17 (251/214)	1.22 (145/118)	1.64 (46/28)	0.61 (26/42)	1.23 (32/26)	**.013**
Mean age of disease onset	68.53 ± 10.8 [37–92.5]	73.79 ± 8.72 [44–92.5]	62.75 ± 9.10 [40–83]	58.85 ± 9.7 [37–77]	63.5 ± 8.9 [44–81]	**<.001**
Patient with age of disease onset <65 years	167 (35.99%)	39 (14.82%)	43 (57.34%)	54 (70.41%)	30 (51.72%)	**<.001**
Patients with age of disease onset ≥65 years	297 (64.01%)	224 (85.17%)	32 (42.67%)	14 (20.59%)	28 (48.27%)	
Mean age of onset of parkinsonism	65.72 ± 19.8 [37–94]	69.67 ± 22.2 [45 –94]	61.24 ± 15.5 [40–83]	57.42 ± 15.4 [37–77]	63.37 ± 12.4 [43–81]	.0859
Patients with age of parkinsonism onset <40 years	2 (0.46%)	0 (0.0%)	0 (0.0)	2 (3.07%)	0 (0.0)	**.031**
Patients with age of parkinsonism onset 40–49 years	20 (4.69%)	1 (0.41%)	5 (6.94%)	9 (13.84%)	5 (8.62%)	
Patients with age of parkinsonism onset 50–70 years	188 (44.13%)	62 (25.61%)	51 (70.83)	47 (72.30%)	38 (65.51%)	
Patients with age of parkinsonism onset ≥70 years	216 (50.70%)	179 (73.96%)	16 (22.23%)	7 (10.76%)	18 (31.03%)	
Mean age of diagnosis (years)	71.76 ± 11.2 [39–94]	77.13 ± 8.39 [45–94]	66.32 ± 11.7 [42–91]	61.56 ± 9.32 [39–78]	66.38 ± 9.38 [45–81]	**.0023**
Mean diagnostic delay (years)^a^	3.40 ± 3.83	3.32 ± 3.19	4.82 ± 5.93	2.54 ± 2.86	2.95 ± 3.67	**<.001**

Abbreviations: APS, atypical parkinsonian syndromes; CBS, corticobasal syndrome; DLB, dementia with Lewy bodies; MSA, multiple system atrophy; PSP, progressive supranuclear palsy.

^a^ Calculated as difference between Age of APS symptom onset and Age of diagnosis.

* Values in bold are significant < .05.

Initial symptoms were essentially nonmotor among DLB subgroup (60.8%) and motor in the other subgroups. Memory disorders were the most frequent manifestation at the onset (29.95%), especially in DLB subgroup (44.48%), followed by gait disorders and parkinsonism (23.27%, 22.41%, respectively). Gait disorders at the onset of the disease were more pronounced in PSP and to a lesser extent MSA subgroups (respectively 58.67% and 41.17; *p* = .0062), while memory disorders were more associated with DLB (*p* = .00175) (Table 2). Later on, during the disease course, the parkinsonism was of late onset (>70 years) in 50.7% of patients, especially in the DLB subgroup (73.96%). Early‐onset parkinsonism (<50 years) was found among 20 patients (4.69%), and two one patients with young‐onset parkinsonism (<40 years) were noted in MSA subgroup (*p* < .001). The earliest onset of parkinsonism was noticed among AMS patients while the oldest was among the PSP patients (*p* = .085) (Table 1).

**TABLE 2:** Clinical features of atypical parkinsonian syndromes

Clinical features	APS *N* = 464	DLB *N* = 263	PSP *N* = 75	MSA *N* = 68	CBS *N* = 58	*p* Value*
**Inaugural symptoms**						
Motor symptoms	215/464 (46.3%)	67/263 (25.5%)	54/75 (66.7%)	56/68 (62.9%)	38/58 (65.5%)	.549
Nonmotor Symptoms	189/464 (40.7%)	160/263 (60.8%)	11/75 (13.6%)	6/68 (6.74%)	12/58 (20.7%)	
Motor and non motor symptoms	60/464 (12.9%)	36/263 (13.7%)	10/75 (12.3%)	6/68 (6.74%)	8/58 (13.8%)	
Parkinsonism	104/464 (22.41%)	43/263 (16.34%)	18/75 (24.0%)	21/68 (30.88%)	22/58 (37.93%)	**.0497**
Gait disorders	108/464 (23.27%)	26/263 (9.89%)	44/75 (58.67%)	28/68 (41.17%)	10/58 (17.24%)	**.0062**
Memory disorders	139/464 (29.95%)	117/263 (44.48%)	8/75 (10.67%)	3/68 (4.41%)	11/58 (18.96%)	**.00175**
Behavioral disorders	109/464 (23.49%)	85/263 (32.31%)	13/75 (17.34%)	4/68 (5.88%)	5/58 (8.62%)	.0952
**Cognitive disorders on examination**						
Memory disorders	287/361 (79.5%)	181/202 (89.6%)	44/55 (80.0%)	28/53 (52.8%)	34/51 (66.7%)	**.0185**
Dysexecutive Syndrome	285/344 (82.84%)	187/195 (95.89%)	42/50 (84.0%)	33/52 (63.46%)	39/47 (82.97%)	.357
Disorientation	153/464 (32.9%)	112/184 (60.8%)	18/54 (33.4%)	8/51 (15.7%)	15/41 (36.6%)	**.012**
Behavioral disorders	292/358 (81.1%)	185/199 (92.9%)	40/56 (71.4%)	31/52 (59.6%)	36/51 (70.6%)	**.035**
Hallucinations	180/362 (49.7%)	155/203 (76.3%)	8/56 (14.3%)	4/52 (7.7%)	13/52 (25.0%)	**.0318**
Aggressiveness	146/360 (40.5%)	104/202 (51.5%)	17/57 (29.8%)	8/51 (15.7%)	17/50 (34.0%)	**.046**
Depression	276/359 (76.9%)	158/200 (79.5%)	42/56 (75.0%)	38/52 (73.1%)	37/51 (72.5%)	.713
Sleep disorders	206/359 (57.4%)	135/200 (67.5%)	33/57 (57.8%)	22/52 (42.3%)	17/50 (35.0%)	.0603

* Values in bold are significant < .05.

Dysexecutive syndrome was the most common finding on neuropsychological assessment across our cohort (82.84%) without significant difference between all subgroups (*p* = .357). Upon evaluation, memory disorders, disorientation as well as behavioral disorders, hallucinations, and aggressiveness were significantly more pronounced among DLB patients and the lowest among MSA patients (respectively *p* = .0185, *p* = .012, *p* = .035, *p* = .0318, and *p* = .046; Table 2).

One third of the patients (33.6%) were born to consanguineous marriage, mainly of first degree (18.62%). Consanguinity rate was significantly different among the subgroups *p* = .037. In fact, we noted that the highest frequency was seen among CBS patients (40.81%), and the lowest was among MSA patients (20.68%). Family history of neurological disorders was found in 242 APS (56.8%): dementia in 36.59%, parkinsonism in 17.71%, and psychiatric disorders in 18.76%. Family history of dementia and psychiatric diseases was more marked in DLB patients' families, while parkinsonism was more commonly found in CBS and PSP families. APS in the same family were found in 8.72% of patients (37 families) with homogeneous phenotype within individual families. These familial forms were divided in 23 DLB, 5 MSA, 5 CBS families, and 4 PSP families including one PSP family with progressive gait freezing (PSP‐PGF; Table 3).

**TABLE 3:** Consanguinity and family history in APS patients

Consanguinity and family history	APS *N* = 464	DLB *N* = 263	PSP *N* = 75	MSA *N* = 68	CBS *N* = 58	*p* value*
Consanguinity rate	132/393 (33.6%)	81/228 (35.52%)	19/58 (32.75%)	12/58 (20.68%)	20/49 (40.81%)	**.037**
Family history of neurological disorders	242/426 (56.80%)	158/253 (62.45%)	29/61 (47.54%)	25/60 (41.67%)	30/52 (57.69%)	
Family history of dementia	157/432 (36.34%)	112/256 (43.75%)	15/61 (24.59%)	14/59 (23.72%)	20/56 (35.71%)	.101
Family history of parkinsonism	76/429 (17.71%)	45/257 (17.5%)	13/61 (21.31%)	10/59 (16.94%)	16/52 (30.76%)	.175
Family history of psychiatric disorders	79/425 (18.58%)	57/249 (22.89)	7/61 (11.47%)	8/59 (13.56%)	7/56 (12.5%)	.216
Families with similar APS cases	37/429 (8.62%)	23/254 (9.05%)	4/60 (6.67%)	5/59 (8.47%)	5/56 (8.92%)	.720

Abbreviations: APS, atypical parkinsonian syndromes; CBS, corticobasal syndrome; DLB, dementia with Lewy bodies; MSA, multiple system atrophy; PSP, progressive supranuclear palsy.

* Values in bold are significant < .05.

## 4 DISCUSSION

Epidemiology of APS in both sub‐Saharan and North African countries including Tunisia remains unknown. To the best of our knowledge, only few studies have been published on APS in Africa. The present study is the largest cohort ever reported in Africa describing the characteristics of APS in a tertiary referral center (Table 4) ( Adebayo et al., 2013; Amoo et al., 2011; Ka et al., 1988; Kacem et al., 2012; Lekoubou et al., 2014; Ndiaye et al., 2011; Ogunniyi et al., 2002; Okubadejo et al., 2010; Radhakrishnan et al., 1988; Regragui et al., 2013; Scrimgeour, 1993; Shehata et al., 2015).

**TABLE 4:** Previous studies/case reports on atypical Parkinsonian syndromes in Africa: Review of the literature

Authors, year of publication	Country	Studied APS	Number of patients	Setting/Design/period of study Epidemiologic and demographic features/Comments
Ka et al. (1988)	Senegal	MSA	34	Hospital cohort; olivocerebellar atrophy
Radhakrishnan et al. (1988)	Libya (Benghazi)	PSP	6	Community study: January 1983 to December 1986 ‐Crude average annual incidence rate/million inhabitants for PSP: 3
Scrimgeour (1993)	Zimbabwe	PSP	1	Case report, 70 year‐old man
Ogunniyi et al. (2002)	Nigeria	DLB	1	Case report, first autopsy‐confirmed case in sub‐Saharan Africa
Okubadejo et al. (2010) *	Nigeria (Lagos, Southern Nigeria)	DLB, PSP, MSA	8	Cross‐sectional study: 1996–2006 Hospital cohort of 124 patients with parkinsonism; 26 patients with secondary parkinsonism: MSA: *n* = 4; DLB: *n* = 3; PSP: *n* = 1
Amoo et al. (2011) *	Nigeria (Abeokuta, south‐western Nigeria)	DLB	3	Cross‐sectional study: 1998–2007 240,294 participants, Hospital cohort of 108 patients with dementia; hospital frequency of DLB: 1.25 per 100,000; Sex: 3 women with DLB
Ndiaye et al. (2011)	Senegal	DLB	1	Cross‐sectional study: 2004–2005, Hospital cohort of 132 patients seen at a memory clinic, 57 with dementia
Kacem et al. (2012)	Tunisia	PSP	1	Case report, man, age of onset: 52 years
Adebayo et al. (2013)	Nigeria	DLB	1	Case report, 67‐year‐old female
Regragui et al. (2013)	Morocco	MSA	17	Cross‐sectional study: January 2007 to December 2010; Hospital cohort of 17 patients with MSA (MSA‐p: 82.4%); sex‐ratio = 3.26; mean age: 55.3 ± 9.2; age of onset: 52 ± 9 years
Shehata et al. (2015)	Egypt (Cairo)	CBS	26	Prospective, open‐label, observational clinical study: April 2008‐December 2013; Hospital cohort of 26 patients with corticobasal syndrome; sex‐ratio = 0.38; mean symptom duration: 34.3 ± 9.3 months (range 24–39 m) and the mean age at initial assessment: 61.3 ± 4.4 years (range 57–66 years)
Our study (2020)	Tunisia	DLB, MSA, PSP, CBS	464	Retrospective cross‐sectional study; Hospital cohort of 464 patients: DLB: *n* = 263, MSA: *n* = 68, PSP: *n* = 75, CBS: *n* = 58; hospital prevalence of APS was 20.6%; sex‐ratio = 1.2; mean age at the onset of the APS = 68.5 years

Abbreviations: CBS, corticobasal syndrome; DLB, dementia with Lewy bodies; MSA, multiple system atrophy; PSP, progressive supranuclear palsy; NS, Not specified.

In this monocentric cohort, the hospital prevalence of APS among parkinsonism was 20.6%. This proportion varies widely in literature due to methodological disparities and differences in inclusion criteria. Nonetheless, the prevalence of APS found in our cohort lies within the range of 15%–25% among the patients with parkinsonism reported in European tertiary referral centers of movement disorders (Mark, 2001). Moreover, the average annual increase of incidence of APS in our cohort of 38.8% per year aligns with recent reports. In fact, population‐based studies estimated that the global prevalence of parkinsonism will considerably increase in the coming decades (Dorsey et al., 2007; Savica et al., 2013). Many reasons may underpin the changes observed in annual incidence of APS over time such as differences in the number of patients seen in our clinic each year. However, other factors may be incriminated seeing the fact that the rise in APS cases (38.8%) outpassed the overall increase of the outpatients' flow in our Department (7.8%) during the same period. In accordance with the literature (Savica et al., 2013), synucleinopathies (71.2%) comprising DLB and MSA were broadly more represented in our series than tauopathies encompassing PSP and CBS (28.8%). DLB was the most prevalent etiology of APS in our series accounting for more than half of the included cases. In fact, DLB is the second cause of degenerative dementia (15%–20%), and parkinsonism accounted for 68%–78% (Bornebroek & Breteler, 2004). However, to our knowledge, no previous study specified the proportion of DLB in APS. This high rate of DLB in our cohort can be explained by a selection bias because of the specificity of recruitment of the cognition unit annexed to the Department of Neurology of Razi hospital. In terms of prevalence, PSP ranked second in our APS cohort. Indeed, it has been recently suggested that the prevalence of PSP was likely to be higher than previous estimates when nonclassical phenotypes are included according to the MDS PSP diagnostic criteria, as performed in our study (Jabbari et al., 2020). MSA with its two major phenotypes, parkinsonian (MSA‐P) and cerebellar (MSA‐C), is generally considered the most common subtype of classical APS (Horvath et al., 2013). It was the third etiology of APS in our cohort accounting for 14.6% of our patients. The relative frequencies of the two forms of MSA vary widely according to the studied populations. In our MSA cohort, only three patients did not yet develop parkinsonism during the study period. MSA‐P are more common in Western countries (68%–82%), as found in our cohort, whereas MSA‐C prevail in eastern countries (67%) (Tison et al., 2000). Along with previous neuro‐epidemic series, CBS was the less frequent APS in our cohort. This finding can be explained by the various conditions underlying corticobasal syndrome only confirmed in the postmortem (Ali & Josephs, 2018; Horvath et al., 2013).

The data on gender differences in the risk of developing APS are conflicting. The higher incidence of APS in male found in our cohort has been reported in most studies of progressive parkinsonism in western populations (Savica et al., 2013). This male prevalence was found in all the subgroups of APS in our series except for MSA. There are conflicting data concerning gender in this subgroup in the literature (Stamelou & Bhatia, 2016; Tison et al., 2000).

In our cohort, average age of onset of parkinsonism was 65.72 years, most belated in DLB (*p* = .031). In our country, this age of onset is older than that previously reported in Parkinson's disease in a large Tunisian cohort of 226 PD, either in patients with LRRK2 G2019S mutation (with an average of 53.8 years) or in G2019S noncarriers (with an average of 57.5 years) (Ben Romdhan et al., 2018). Moreover, we noticed a predominance of late‐onset parkinsonism (>70 years) in our cohort. Hence, late‐onset parkinsonism is the first feature to consider in etiological orientation. Patients starting their symptoms after the age of 85 years were exclusively of the DLB subgroup, in accordance with the literature (Bornebroek & Breteler, 2004). Conversely, an onset before the age of 65 years was noted in third of the patients in our series, earlier in MSA as previously reported (Tison et al., 2000).

In addition to parkinsonism, other motor and nonmotor features were variably present and associated at the onset or during disease course in the different APS subgroups. Among the motor inaugural features, gait disorders were mostly suggestive of PSP and to lesser extent MSA in our cohort. In fact, the temporal onset of falls was found to be shortest in PSP (6 months), intermediate in MSA, DLB and CBS (from 2 to 4 years), and longest in PD (118 months) (Bhidayasiri et al., 2019). Moreover, recent studies emphasized the high discriminative power of postural instability and gait disorders in differentiating PD from APS supporting gait disorders as substantial diagnostic target (Gaßner et al., 2019). As expected, cognitive, mood, and behavioral disorders were more marked in the DLB subgroup and less common in the MSA subgroup in our cohort except for depression found at similar rates across all APS subgroups. Along with these findings, in a recent systematic review by Belvisi et al. (2018) on neuropsychiatric disturbances in APS, MSA patients showed a higher frequency of depressive disorders when compared to healthy controls. Mood disturbances in MSA patients correlated with deficits in cognitive performances, highlighting a possible pathophysiological link between behavioral/psychiatric disturbances and frontal cognitive in patients with MSA (Belvisi et al., 2018). Hence, the integration of these motor, nonmotor, and non‐neurological symptoms according a pragmatic approach and the recognition of multiple red flags in an individual patient may increase the likelihood of APS (Bhidayasiri et al., 2019; Mulroy et al., 2019).

Family history of parkinsonism was found in 17.71%, with no difference between subgroups. Although only family history of Parkinson's disease is considered as a risk factor for APS in the literature (Boot et al., 2013), dementia and psychiatric disorders were more frequent in families of our DLB patients. In fact, in a study of Boot et al., assessing risk factors in 147 DLB, it was established that they were a mix of those of PD and Alzheimer's disease (Boot et al., 2013), with a stronger association in DLB. Furthermore, in our series, we identified 37 families of APS. Familial aggregation was reported in all APS. The contribution of genetic factors to atypical parkinsonian syndromes is increasingly recognized (Scholz & Bras, 2015). In parallel, there is a growing body of evidence on new genetic conditions presenting with features of APS, called “atypical” atypical parkinsonism (Giagkou et al., 2019; Stamelou et al., 2013). To establish whether these familial cases of APS are “true” APS or AP‐look‐alikes would require further genetic and pathological investigations.

Some limitations should be kept in mind though regarding the findings of this study and warrant analysis. Many factors of bias inherent to the selectivity of patients in a specialized center prevent the simple extrapolation of our findings to the general Tunisian population. Another major limitation of our study is the lack of neuropathological confirmation. The fact that our cohort comprised only clinically diagnosed patients exposes to the risk of diagnostic uncertainty because of heterogeneous clinical presentations of APS and possible presence of APS‐look‐alikes. Nevertheless, in our particular context and mainly because of cultural believes, the construction of autopsy‐confirmed banks of APS seems to be a difficult task not to be solved soon.

## 5 CONCLUSION

This study provides preliminary epidemiological data on APS in Tunisia through findings from a monocentric tertiary referral institution with a review on previously reported studies on APS in Africa. Despite the limitations of our study, this initial research sheds lights on the demographic and clinical peculiarities of the different etiological subgroups of APS setting the stage for a diagnosis strategy adjusted to our context. Further epidemiologic studies would also help to understand potential ethnic and genetic singularities of APS in these regions.

## CONFLICTS OF INTEREST

None of the authors have any conflicts of interest to disclose.

## AUTHOR CONTRIBUTION

In keeping with the latest guidelines of the International Committee of Medical Journal Editors, Dr Amina NASRI contributed to the conception and design of the study, drafting of the manuscript, analysis and interpretation of data; Pr Mouna BEN DJEBARA contributed to the conception and design of the study and to the interpretation of data for the work, Dr Ikram SGHAIER contributed to the acquisition of data and statistical analysis, Dr Saloua MRABET and Dr Sabrina ZIDI contributed to the acquisition and analysis of data for the work; Pr Amina GARGOURI and Pr Imen KACEM contributed to revising the manuscript critically for important intellectual content; and Pr Riadh GOUIDER contributed to the conception and design of the study and gave final approval of the version to be published.

### Peer Review

The peer review history for this article is available at https://publons.com/publon/10.1002/brb3.1924.

## Data Availability

The anonymized data that support the findings of this study are available on request from the first author.

## References

[brb31924-bib-0001] Adebayo, P. B. , Ajani, A. A. , Adeniji, O. A. , & Akinyemi, R. O. (2013). Neuropsychiatric and parkinsonian manifestations of dementia: A case report in a Nigerian woman. Annals of African Medicine, 12(1), 46 10.4103/1596-3519.108252 23480996

[brb31924-bib-0002] Ali, F. , & Josephs, K. A. (2018). Corticobasal degeneration: Key emerging issues. Journal of Neurology, 265(2), 439–445. 10.1007/s00415-017-8644-3 29063240

[brb31924-bib-0003] Amoo, G. , Akinyemi, R. O. , Onofa, L. U. , Akinyemi, J. O. , Baiyewu, O. , Ogunlesi, A. O. , & Ogunniyi, A. (2011). Profile of clinically‐diagnosed dementias in a neuropsychiatric practice in Abeokuta, South‐Western Nigeria. African Journal of Psychiatry, 14(5), 377–382. 10.4314/ajpsy.v14i5.5 22183468

[brb31924-bib-0004] Armstrong, M. J. , Litvan, I. , Lang, A. E. , Bak, T. H. , Bhatia, K. P. , Borroni, B. , Boxer, A. L. , Dickson, D. W. , Grossman, M. , Hallett, M. , Josephs, K. A. , Kertesz, A. , Lee, S. E. , Miller, B. L. , Reich, S. G. , Riley, D. E. , Tolosa, E. , Troster, A. I. , Vidailhet, M. , & Weiner, W. J. (2013). Criteria for the diagnosis of corticobasal degeneration. Neurology, 80(5), 496–503. 10.1212/WNL.0b013e31827f0fd1 23359374 PMC3590050

[brb31924-bib-0005] Arnao, V. , Gangitano, M. , Giacalone, F. , Riolo, M. , Savettieri, G. , & Aridon, P. (2015). Corticobasal syndrome‐like variant of Creutzfeldt‐Jakob disease: Clinical description of two cases. Neurological Sciences, 36(7), 1303–1305. 10.1007/s10072-014-2043-7 25515788

[brb31924-bib-0006] Belvisi, D. , Berardelli, I. , Suppa, A. , Fabbrini, A. , Pasquini, M. , Pompili, M. , & Fabbrini, G. (2018). Neuropsychiatric disturbances in atypical parkinsonian disorders. Neuropsychiatric Disease and Treatment, 14, 2643 10.2147/NDT.S178263 30349262 PMC6186304

[brb31924-bib-0007] Ben Romdhan, S. , Farhat, N. , Nasri, A. , Lesage, S. , Hdiji, O. , Ben Djebara, M. , Landoulsi, Z. , Stevanin, G. , Brice, A. , Damak, M. , Gouider, R. , & Mhiri, C. (2018). LRRK2 G2019S Parkinson's disease with more benign phenotype than idiopathic. Acta Neurologica Scandinavica, 138(5), 425–431. 10.1111/ane.12996 29989150

[brb31924-bib-0008] Bhidayasiri, R. , Sringean, J. , Reich, S. G. , & Colosimo, C. (2019). Red flags phenotyping: A systematic review on clinical features in atypical parkinsonian disorders. Parkinsonism & Related Disorders, 59, 82–92. 10.1016/j.parkreldis.2018.10.009 30409560

[brb31924-bib-0009] Boot, B. P. , Orr, C. F. , Ahlskog, J. E. , Ferman, T. J. , Roberts, R. , Pankratz, V. S. , Dickson, D. W. , Parisi, J. , Aakre, J. A. , Geda, Y. E. , Knopman, D. S. , Petersen, R. C. , & Boeve, B. F. (2013). Risk factors for dementia with Lewy bodies: A case‐control study. Neurology, 81(9), 833–840. 10.1212/WNL.0b013e3182a2cbd1 23892702 PMC3908463

[brb31924-bib-0010] Bornebroek, M. , & Breteler, M. M. (2004). Epidemiology of non‐AD dementias. Clinical Neuroscience Research, 3(6), 349–361. 10.1016/j.cnr.2004.04.002

[brb31924-bib-0011] Deutschländer, A. B. , Ross, O. A. , Dickson, D. W. , & Wszolek, Z. K. (2018). Atypical Parkinsonian syndromes: A general neurologist's perspective. European Journal of Neurology, 25(1), 41–58. 10.1111/ene.13412 28803444 PMC7646945

[brb31924-bib-0012] Dorsey, E. R. , Constantinescu, R. , Thompson, J. P. , Biglan, K. M. , Holloway, R. G. , Kieburtz, K. , Marshall, F. J. , Ravina, B. M. , Schifitto, G. , Siderowf, A. , & Tanner, C. M. (2007). Projected number of people with Parkinson disease in the most populous nations, 2005 through 2030. Neurology, 68(5), 384–386. 10.1212/01.wnl.0000247740.47667.03 17082464

[brb31924-bib-0013] Foguem, C. , & Kamsu‐Foguem, B. (2016). Neurodegeneration in tauopathies and synucleinopathies. Revue Neurologique, 172(11), 709–714. 10.1016/j.neurol.2016.05.002 27344208

[brb31924-bib-0014] Gaßner, H. , Raccagni, C. , Eskofier, B. M. , Klucken, J. , & Wenning, G. K. (2019). The diagnostic scope of sensor‐based gait analysis in atypical Parkinsonism: Further observations. Frontiers in Neurology, 10, 5 10.3389/fneur.2019.00005 30723450 PMC6349719

[brb31924-bib-0015] Giagkou, N. , Bhatia, K. P. , Höglinger, G. U. , & Stamelou, M. (2019). Genetic mimics of the non‐genetic atypical parkinsonian disorders–the ‘atypical’atypical. International Review of Neurobiology, 149, 327–351. 10.1016/bs.irn.2019.10.008 31779819

[brb31924-bib-0016] Gilman, S. , Wenning, G. K. , Low, P. A. , Brooks, D. J. , Mathias, C. J. , Trojanowski, J. Q. , Wood, N. W. , Colosimo, C. , Durr, A. , Fowler, C. J. , Kaufmann, H. , Klockgether, T. , Lees, A. , Poewe, W. , Quinn, N. , Revesz, T. , Robertson, D. , Sandroni, P. , Seppi, K. , & Vidailhet, M. (2008). Second consensus statement on the diagnosis of multiple system atrophy. Neurology, 71(9), 670–676. 10.1212/01.wnl.0000324625.00404.15 18725592 PMC2676993

[brb31924-bib-0017] Höglinger, G. U. , Respondek, G. , Stamelou, M. , Kurz, C. , Josephs, K. A. , Lang, A. E. , Mollenhauer, B. , Müller, U. , Nilsson, C. , Whitwell, J. L. , Arzberger, T. , Englund, E. , Gelpi, E. , Giese, A. , Irwin, D. J. , Meissner, W. G. , Pantelyat, A. , Rajput, A. , van Swieten, J. C. , … Litvan, I. (2017). Clinical diagnosis of progressive supranuclear palsy: The movement disorder society criteria. Movement Disorders, 32(6), 853–864. 10.1002/mds.26987 28467028 PMC5516529

[brb31924-bib-0018] Horvath, J. , Burkhard, P. R. , Bouras, C. , & Kövari, E. (2013). Etiologies of Parkinsonism in a century‐long autopsy‐based cohort. Brain Pathology, 23(1), 28–33. 10.1111/j.1750-3639.2012.00611 22702335 PMC8029343

[brb31924-bib-0019] Hussein, S. , & Ismail, M. (2017). Ageing and elderly care in the Arab Region: Policy challenges and opportunities. Ageing International, 42(3), 274–289. 10.1007/s12126-016-9244-8 28890585 PMC5569126

[brb31924-bib-0020] Jabbari, E. , Holland, N. , Chelban, V. , Jones, P. S. , Lamb, R. , Rawlinson, C. , Guo, T. , Costantini, A. A. , Tan, M. M. X. , Heslegrave, A. J. , Roncaroli, F. , Klein, J. C. , Ansorge, O. , Allinson, K. S. J. , Jaunmuktane, Z. , Holton, J. L. , Revesz, T. , Warner, T. T. , Lees, A. J. , … Morris, H. R. (2020). Diagnosis across the spectrum of progressive supranuclear palsy and corticobasal syndrome. JAMA Neurology, 77(3), 377–387. 10.1001/jamaneurol.2019.4347 31860007 PMC6990759

[brb31924-bib-0021] Ka, M. , Kone, S. , Ndiaye, M. , & Ndiaye, I. P. (1988). Olivocerebellar atrophy predominantly affecting the vermis (clinical and etiopathogenic aspects apropos of 34 cases). Dakar Medical, 33(1–4), 30 10.3349/ymj.1988.29.3.233 3079273

[brb31924-bib-0022] Kacem, I. , Gargouri, A. , Ben‐Djebara, M. , & Gouider, R. (2012). Clinical and single‐photon emission computed tomography study of pure akinesia with freezing of gait. Neurosciences, 17(1), 66–68. 22246015

[brb31924-bib-0023] Koga, S. , Roemer, S. F. , Kasanuki, K. , & Dickson, D. W. (2019). Cerebrovascular pathology presenting as corticobasal syndrome: An autopsy case series of “vascular CBS”. Parkinsonism & Related Disorders, 68, 79–84. 10.1016/j.parkreldis.2019.09.001 31621626 PMC7141792

[brb31924-bib-0024] Lekoubou, A. , Echouffo‐Tcheugui, J. B. , & Kengne, A. P. (2014). Epidemiology of neurodegenerative diseases in sub‐Saharan Africa: A systematic review. BMC Public Health, 14(1), 653 10.1186/1471-2458-14-653 24969686 PMC4094534

[brb31924-bib-0025] Mark, M. H. (2001). Lumping and splitting the Parkinson Plus syndromes: Dementia with Lewy bodies, multiple system atrophy, progressive supranuclear palsy, and cortical‐basal ganglionic degeneration. Neurologic Clinics, 2001(19), 607–627. 10.1016/s0733-8619(05)70037-2 11532646

[brb31924-bib-0026] McKeith, I. G. , Boeve, B. F. , Dickson, D. W. , Halliday, G. , Taylor, J.‐P. , Weintraub, D. , Aarsland, D. , Galvin, J. , Attems, J. , Ballard, C. G. , Bayston, A. , Beach, T. G. , Blanc, F. , Bohnen, N. , Bonanni, L. , Bras, J. , Brundin, P. , Burn, D. , Chen‐Plotkin, A. , … Kosaka, K. (2017). Diagnosis and management of dementia with Lewy bodies: Fourth consensus report of the DLB Consortium. Neurology, 89(1), 88–100. 10.1212/WNL.0000000000004058 28592453 PMC5496518

[brb31924-bib-0027] Morelli, M. , Fera, F. , Bono, F. , Fratto, A. , Arabia, G. , & Quattrone, A. (2014). Intraventricular tumor presenting as progressive supranuclear palsy–like phenotype. Neurology, 83(10), 948 10.1212/WNL.0000000000000748 25179998

[brb31924-bib-0028] Mulroy, E. , Stamelou, M. , & Bhatia, K. (2019). How to approach a patient with parkinsonism – red flags for atypical parkinsonism. International Review of Neurobiology, 149, 1–34. 10.1016/bs.irn.2019.10.001 31779810

[brb31924-bib-0029] Ndiaye, D. , Sylla, A. , Toure, K. , Thiam, M. H. , & Gueye, M. (2011). Assessment of a Senegalese memory clinic. AJNS, 30(1), 1–9.

[brb31924-bib-0030] Ogunniyi, A. , Akang, E. E. U. , Gureje, O. , Takao, M. , Piccardo, P. , Baiyewu, O. , Hall, K. S. , Ghetti, B. , & Hendrie, H. C. (2002). Dementia with Lewy bodies in a Nigerian: A case report. International Psychogeriatrics, 14(2), 211–218. 10.1017/s1041610202008402 12243211

[brb31924-bib-0031] Okubadejo, N. U. , Ojo, O. O. , & Oshinaike, O. O. (2010). Clinical profile of parkinsonism and Parkinson's disease in Lagos, Southwestern Nigeria. BMC Neurology, 10(1), 1 10.1186/1471-2377-10-1 20051133 PMC2806862

[brb31924-bib-0032] Radhakrishnan, K. , Thacker, A. K. , Maloo, J. C. , Gerryo, S. E. , & Mousa, M. E. (1988). Descriptive epidemiology of some rare neurological diseases in Benghazi, Libya. Neuroepidemiology, 7(3), 159–164. 10.1159/000110150 3405368

[brb31924-bib-0033] Regragui, W. , Lachhab, L. , Razine, R. , Benjelloun, H. , Benhaddou, E. A. , Benomar, A. , & Yahyaoui, M. (2013). Profile of multiple system atrophy in Moroccan patients attending a movement disorders outpatient clinic in Rabat university hospital. Revue Neurologique, 169(2), 121–125. 10.1016/j.neurol.2012.04.006 22763206

[brb31924-bib-0034] Savica, R. , Grossardt, B. R. , Bower, J. H. , Ahlskog, J. E. , & Rocca, W. A. (2013). Incidence and pathology of synucleinopathies and tauopathies related to parkinsonism. JAMA Neurology, 70(7), 859–866. 10.1001/jamaneurol.2013.114 23689920 PMC3707980

[brb31924-bib-0035] Scholz, S. W. , & Bras, J. (2015). Genetics underlying atypical parkinsonism and related neurodegenerative disorders. International Journal of Molecular Sciences, 16(10), 24629–24655. 10.3390/ijms161024629 26501269 PMC4632769

[brb31924-bib-0036] Scrimgeour, E. M. (1993). Progressive supranuclear palsy in a Zimbabwean man. West African Journal of Medicine, 12(3), 175. 8312218

[brb31924-bib-0037] Shehata, H. S. , Shalaby, N. M. , Esmail, E. H. , & Fahmy, E. (2015). Corticobasal degeneration: Clinical characteristics and multidisciplinary therapeutic approach in 26 patients. Neurological Sciences, 36(9), 1651–1657. 10.1016/s0733-8619(05)70037-2 25917399

[brb31924-bib-0038] Stamelou, M. , & Bhatia, K. P. (2016). Atypical parkinsonism – new advances. Current Opinion in Neurology, 29(4), 480–485. 10.1097/WCO.0000000000000355 27272977

[brb31924-bib-0039] Stamelou, M. , Quinn, N. P. , & Bhatia, K. P. (2013). “Atypical” atypical parkinsonism: New genetic conditions presenting with features of progressive supranuclear palsy, corticobasal degeneration, or multiple system atrophy—a diagnostic guide. Movement Disorders, 28(9), 1184–1199. 10.1002/mds.25509 23720239

[brb31924-bib-0040] Tison, F. , Yekhlef, F. , Chrysostome, V. , & Sourgen, C. (2000). Prevalence of multiple system atrophy. Lancet, 355(9202), 495–496. 10.1016/S0140-6736(00)82050-4 10841152

